# CopyVAE: a variational autoencoder-based approach for copy number variation inference using single-cell transcriptomics

**DOI:** 10.1093/bioinformatics/btae284

**Published:** 2024-04-27

**Authors:** Semih Kurt, Mandi Chen, Hosein Toosi, Xinsong Chen, Camilla Engblom, Jeff Mold, Johan Hartman, Jens Lagergren

**Affiliations:** School of EECS and SciLifeLab, KTH Royal Institute of Technology, Stockholm, 100 44, Sweden; School of EECS and SciLifeLab, KTH Royal Institute of Technology, Stockholm, 100 44, Sweden; School of EECS and SciLifeLab, KTH Royal Institute of Technology, Stockholm, 100 44, Sweden; Department of Oncology and Pathology, Karolinska Institutet, Solna, 171 77, Sweden; Department of Cell and Molecular Biology, Karolinska Institutet, Solna, 171 77, Sweden; Department of Cell and Molecular Biology, Karolinska Institutet, Solna, 171 77, Sweden; Department of Oncology and Pathology, Karolinska Institutet, Solna, 171 77, Sweden; Department of Clinical Pathology and Cytology, Karolinska University Laboratory, Solna, 171 76, Sweden; School of EECS and SciLifeLab, KTH Royal Institute of Technology, Stockholm, 100 44, Sweden

## Abstract

**Motivation:**

Copy number variations (CNVs) are common genetic alterations in tumour cells. The delineation of CNVs holds promise for enhancing our comprehension of cancer progression. Moreover, accurate inference of CNVs from single-cell sequencing data is essential for unravelling intratumoral heterogeneity. However, existing inference methods face limitations in resolution and sensitivity.

**Results:**

To address these challenges, we present CopyVAE, a deep learning framework based on a variational autoencoder architecture. Through experiments, we demonstrated that CopyVAE can accurately and reliably detect CNVs from data obtained using single-cell RNA sequencing. CopyVAE surpasses existing methods in terms of sensitivity and specificity. We also discussed CopyVAE’s potential to advance our understanding of genetic alterations and their impact on disease advancement.

**Availability and implementation:**

CopyVAE is implemented and freely available under MIT license at https://github.com/kurtsemih/copyVAE.

## 1 Introduction

Copy number variations (CNVs), which refer to deletions and duplications of specific genomic regions, play a crucial role in maintaining normal cellular function and contribute to disease development. CNVs encompass duplications (additional copies of sequence) and deletions (losses of genetic material) and typically occur at an intermediate scale, with segments ranging from >1000 base pairs to <5 megabases (Eichler 2008). These genetic changes have been implicated in various disorders, including cancer, neurological diseases, and cardiovascular diseases. CNV patterns vary among tumour and normal samples, various tumour subtypes, cancer patients with different drug responses, and varying survival times, making it a significant and robust biomarker distinct from gene expression, which can be easily disrupted and displays randomness (Huang 2019). In a previous study (Beroukhim *et al.* 2010), 158 specific regions of somatic CNVs that were significantly different in various types of cancer were found. These regions include 75 700 copy number gains and 55 101 copy number losses, which were observed across the analysed 3131 cancers. More recent research (Harbers *et al.* 2021) analysing TCGA datasets has revealed that somatic copy number amplifications or deletions longer than 10 kb are observed in 87.5% of tumour types. Accurate detection of CNVs has significant implications for understanding disease mechanisms and designing targeted therapies.

Numerous tools have been developed to detect CNVs using next-generation sequencing (NGS) data. These include a wide range of methods applicable to whole-genome sequencing (WGS) data (Abyzov *et al.* 2011, Boeva *et al.* 2011, Miller *et al.* 2011, Talevich *et al.* 2016, Dharanipragada *et al.* 2018, Zhang *et al.* 2022) and whole-exome sequencing (WES) data (Krumm *et al.* 2012, Backenroth *et al.* 2014, Jiang *et al.* 2015, Bigio *et al.* 2021). Furthermore, several efforts (Özden *et al.* 2022, Rapti *et al.* 2022) have been made to combine WGS and WES data to enhance the accuracy of CNV detection. In cancer studies, methods utilizing single-cell genome sequencing (scDNA-seq) and single-cell transcriptome sequencing (scRNA-seq) data have emerged as valuable tools for gaining deeper insights into intratumoral heterogeneity. Existing methods (Shah *et al.* 2006, Nilsen *et al.* 2012, Garvin *et al.* 2015, Wang *et al.* 2020, Zaccaria and Raphael 2021) developed for detecting CNVs in scDNA-seq data provide a high-resolution inference of absolute copy numbers, which represents the number of copies of a specific genomic region within a single cell. However, these tools are often affected by scDNA-seq library preparation (Mallory *et al.* 2020), and computational inefficiency arises specifically from the large size of scDNA-seq data. In addition, acquiring such data in cancer studies can be challenging because DNA-seq data are usually deposited in raw format and often restricted access as opposed to publicly available expression data that come with RNA-seq.

CNVs exhibit a high correlation with gene expression (Shao *et al.* 2019), making them possible to infer from expression levels. Consequently, there are tools specifically designed for CNV calling in scRNA-seq data. However, this approach faces significant challenges attributed to the coexistence of immune and tumour cells in the tumour microenvironment and the presence of other factors regulating transcript abundance (e.g. cell-cycle state). There have been previous approaches in the literature to address these challenges and estimate genomic copy number profiles from scRNA-seq data. InferCNV (Patel *et al.* 2014), needs the classification of normal cells in the tumour microenvironment in advance. Another method, SCEVAN (De Falco *et al.* 2023), detects normal cells by the use of a set of gene signatures from public collections (Yoshihara *et al.* 2013, Caruso *et al.* 2020). A recent approach, Numbat (Gao *et al.* 2023), also uses cell type or tumour-specific markers to distinguish between malignant and nonmalignant cells. It also requires alignment files in addition to the expression matrix in order to extract allelic read counts in the preprocessing steps. All these methods require either additional information or a priori knowledge of cell types and gene signatures to estimate the copy number profiles of tumour cells. An automated approach to overcome these restrictions is proposed by CopyKAT (Gao *et al.* 2021). CopyKAT performs a two-step classification of normal and tumour cells. The first step utilizes normalized gene expression counts to identify confident normal cells whereas the second step uses inferred copy number profiles to make the final classification of normal and tumour cells. With this approach, a misclassification in the first step may lead to a much larger misclassification in the final step. CopyKAT obtains copy number profiles via Markov chain Monte Carlo (MCMC) segmentation and Kolmogorov–Smirnov (KS) tests. Although CopyKAT is an automated approach, it is computationally heavy and its performance may be affected by the sensitivity of the classification steps.

Here, we present CopyVAE, a variational autoencoder (VAE)-based approach for accurate CNV inference using scRNA-seq data. CopyVAE automatically detects normal and tumour cells in a given cell population, and estimates the copy number profiles of the tumour cells. It has a straight-forward pipeline based on a small VAE, and does not require cell-type specific gene signatures, tumour-specific markers, or any form of prior information.

This paper is organized as follows. First, we provide an explanation of CopyVAE and its underlying principles in the Section 2. Next, we present the experimental setup, including evaluation metrics and the datasets used, as well as the results and performance evaluation of CopyVAE in the Section 3, followed by a discussion of the implications and potential applications of our findings. Finally, we conclude with a summary of the key contributions and future directions of this research.

## 2 Materials and methods

### 2.1 Data preprocessing

The gene expression counts from single cells are initially annotated and sorted according to their genomic positions. To ensure data quality, a quality control step is performed, which involves removing low-quality cells and nonexpressed genes. The count matrix is then filtered based on data quality criteria, excluding mitochondrially encoded genes, HLA genes, and cell cycle genes (Macosko *et al.* 2015). In addition, the cells are normalized by their total counts across all genes, so that every cell has the same total count after normalization. To facilitate analysis and reduce the effect of dropouts, the genes are grouped into bins with 25 genes as done in Gao *et al.* (2021) (see Supplementary Section S3.1.1 for further motivation). The mean count value for each bin is computed, resulting in a count matrix with dimensions of cell × bin. This matrix, which represents the input for CopyVAE, is used for subsequent analyses.

### 2.2 CopyVAE workflow

**Figure 1.**
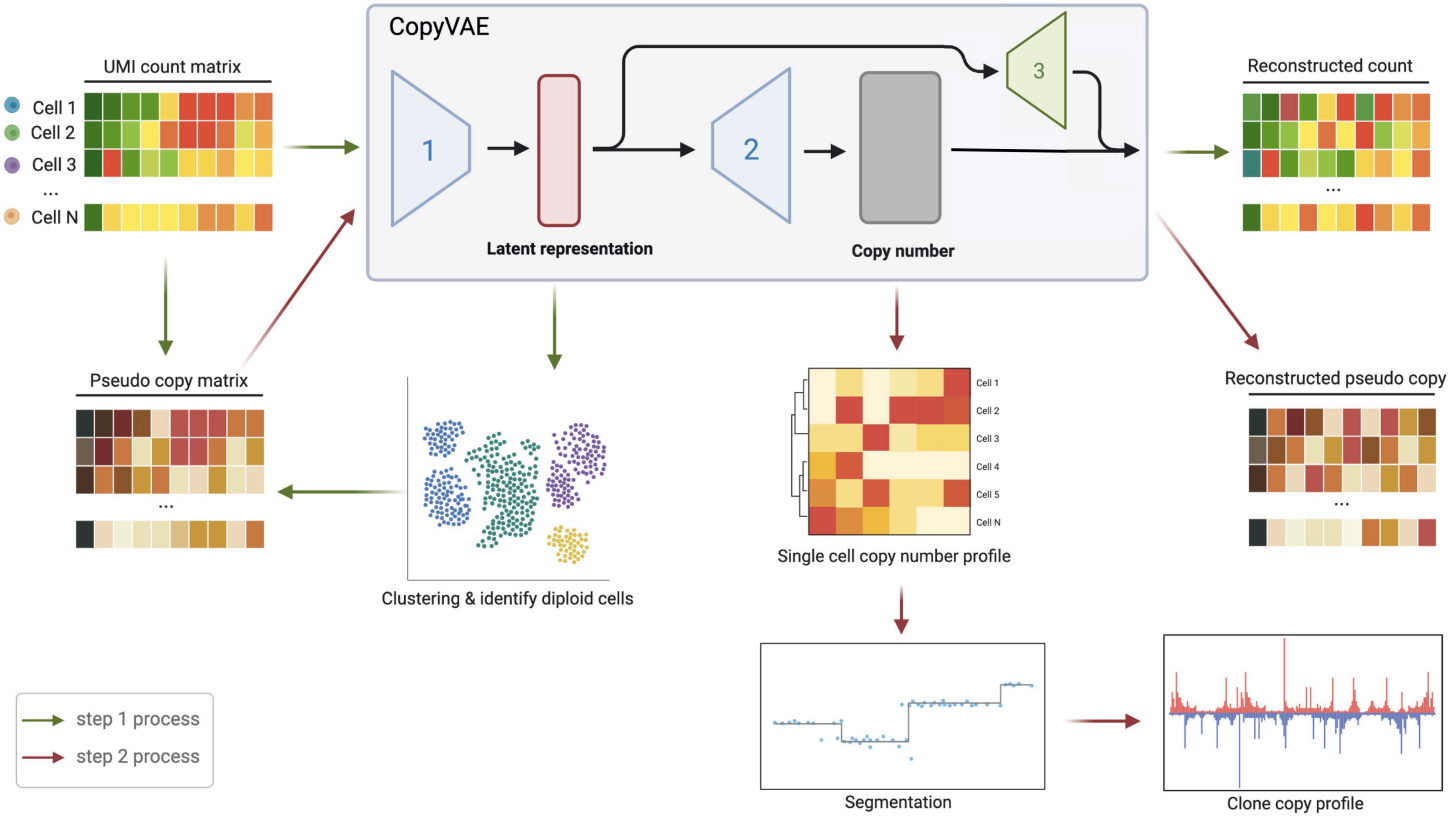
CopyVAE workflow: Step 1: CopyVAE takes count matrix as input and is trained to learn latent representations for cells. Diploid cells are identified using k-means clustering and auto-correlation comparison. The baseline expression levels are calculated from the expression profiles of identified diploid cells, and a pseudo copy matrix is generated for approximate copy number estimation. Step 2: CopyVAE takes pseudo copy matrix as input and is trained to refine copy number estimation, followed by a likelihood-based segmentation algorithm to integrate copy number profiles within aneuploid clones and call breakpoints individually for each clone.

The CopyVAE workflow (Fig. 1) consists of two steps: (i) estimation of the baseline gene expression profile of diploid cells, and (ii) inference of copy numbers for all cells.

In step 1, CopyVAE is trained on the count matrix to learn the latent representation of each cell. These representations are clustered via k-means clustering method and the auto-correlation score is computed for each cluster. Based on the assumption that normal cells exhibit lesser diversity in contrast to tumour cells (R Gao *et al.* 2021), the cluster with the greatest auto-correlation is identified as the diploid cells. The expression profiles of the identified diploid cells are aggregated to serve as the baseline expression profile. The count matrix is normalized by this baseline profile to obtain a pseudo copy matrix, which provides preliminary estimates of the copy numbers of bins in all cells.

In step 2, CopyVAE is retrained using the pseudo copy matrix as input, in order to learn a more refined estimation of the copy number of each bin in each cell. Based on these estimates, a likelihood-based segmentation algorithm (Jackson *et al.* 2005, Killick *et al.* 2012) is used to determine the breakpoints for each tumour clone individually. Single-cell copy numbers are obtained by computing the copy number of each segment between two breakpoints as the median value of the bin copy numbers within that segment. The single-cell copy number profiles are then aggregated by calculating the mean value of the segment copy numbers, resulting in the clone copy number profile.

Further details can be found in Supplementary Section S1.

## 3 Experiments and results

We began our experiments with a synthetic dataset, where the count matrix was generated using known single-cell copy number profiles. Subsequently, we benchmarked copyVAE against state-of-the-art methods using two breast cancer datasets. In this context, the inferred copy number profiles obtained from bulk DNA sequencing and whole-exome sequencing analysis methods served as ground truth reference. CopyVAE generates copy number calls for each gene, which are then mapped to corresponding genomic positions to derive the positional copy numbers. For genomic positions that do not align with any called genes, the copy numbers are approximated using the nearest called position. The evaluated positions are determined based on the ground truth profiles.

### 3.1 Evaluation metrics

To quantify the accuracy of the estimated copy numbers, we calculate the Pearson correlation and cosine similarity between the inferred and the ground truth copy number profiles. The Pearson correlation coefficient ranges from −1 to 1, with 0 indicating no correlation, 1 representing a perfect positive correlation, and −1 indicating a perfect negative correlation. Likewise, the cosine similarity ranges from −1 to 1, with 1 indicating that the vectors are proportional, 0 indicating orthogonality, and −1 indicating opposite directions. In addition, we calculated the average positional Manhattan distance and average positional Euclidean distance between the estimated and the ground truth copy number profiles. The average positional distances are calculated via normalizing the total distances by the number of genomic positions. By doing so, we obtain comparable measurements between different samples.

### 3.2 Experiments on synthetic data

#### 3.2.1 Synthetic data generation

An important component of our analysis is synthetic data where ground truth copy number values are available, which allows a true quantitative evaluation of our method. The synthetic data generation process involves two main steps: generating synthetic data for normal diploid cells and introducing synthetic CNVs. A similar methodology was adopted by De Falco *et al.* (2023) in their synthetic data generation process.

We initially generate a normal diploid cell matrix using a deep generative model called scVI (Lopez *et al.* 2018), based on a real scRNA-seq dataset obtained from bone marrow aspirates [referred to as BM dataset, Yao *et al.* (2023)]. ScVI is a deep generative model with the capacity to learn its parameters from real data and generate synthetic data that shares common features with the provided real data. To do that, it learns the latent representation of each cell via an encoder and maps these latent representations to a gene-count matrix via a decoder (Kingma and Welling 2013, Kingma and Ba 2014, Lopez *et al.* 2018). We train a scVI model with the normal cells from the BM dataset, and obtain the latent representations. Then, we infer the distribution of the latent representation via maximum likelihood. Next, we sample synthetic latent vectors from the inferred distribution and pass them to the trained decoder. The output of the decoder constitutes our synthetic matrix corresponding to normal diploid cells.

Next, we introduce synthetic CNVs on the previously obtained matrix of synthetic normal diploid cells, to have synthetic aneuploid cells. We choose 80% of total cells to be aneuploid. We consider integer copy numbers ranging from 0 to 6, and categorize 0, 1 as loss whereas 3, 4, 5, 6 as gain. We randomly choose the locations and types (loss or gain) of CNVs. These are shared by all synthetic aneuploid cells. However, to ensure aneuploid cell diversity, the actual copy numbers for loss and gain regions in each aneuploid cell are chosen independently. After getting the copy number profile of each aneuploid cell, we modify the corresponding rows of the synthetic normal diploid cell matrix to get the final synthetic data. We generated 10 different synthetic datasets this way, naming them BM2-v0s0 through BM2-v0s9.

#### 3.2.2 Copy number estimation on synthetic data

**Figure 2.**
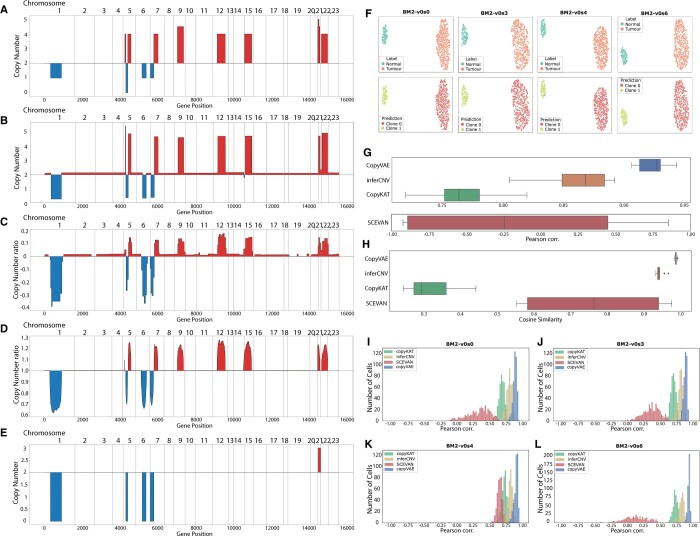
Performance evaluation on synthetic data: (A) ground truth copy number profile of BM2-v0s0 sample. (B) BM2-v0s0 copy number profile estimated by CopyVAE. (C) BM2-v0s0 copy number profile estimated by CopyKat. (D) BM2-v0s0 copy number profile estimated by inferCNV. (E) BM2-v0s0 copy number profile estimated by SCEVAN. (F) CopyVAE clusters (bottom) versus ground truth clusters (top) demonstrated in the UMAP projection of latent representations. (G) Boxplots of Pearson correlation between ground truth tumour clone copy number profile and estimation in all samples. (H) Boxplots of Cosine similarity between ground truth tumour clone copy number profile and estimation in all samples. (I) Pearson correlation of single cells in sample BM2-v0s0. (J) Pearson correlation of single cells in sample BM2-v0s3. (K) Pearson correlation of single cells in sample BM2-v0s4. (L) Pearson correlation of single cells in sample BM2-v0s6.

Using synthetic datasets, we conducted an empirical evaluation of CopyVAE in comparison to the existing methods: CopyKat, inferCNV, and SCEVAN (Fig. 2A–F). Notably, Numbat was excluded from this experiment due to its prerequisite for BAM files containing SNP information.

We first investigated the results of clonal copy number profile estimation (Fig. 2G and H). CopyVAE demonstrated the highest Pearson correlation coefficient (from 0.906 to 0.944) and cosine similarity (from 0.981 to 0.991) to the ground truth profile in all ten samples, thus exhibiting superior performance over the other methods evaluated (Supplementary Section S2). Furthermore, InferCNV and CopyKat displayed consistent performance with an average Pearson correlation coefficient of 0.86 and 0.77 (Fig. 2G), respectively. SCEVAN yielded a Pearson correlation coefficient of 0.84 and 0.85 in only two of the ten samples and yielded low coefficients (ranging from −0.92 to 0.46) in the remaining eight samples, resulting in a high variance in overall performance (Fig. 2G). CopyKat demonstrated the lowest cosine similarity to the ground truth profile in all 10 samples (Fig. 2H, Supplementary Section S2).

Subsequently, we proceeded to assess the accuracy of copy number estimation at the single-cell level by computing the Pearson correlation coefficient between the estimated and ground truth copy numbers for each tumour cell. The results demonstrated that the single-cell copy number profiles generated by CopyVAE exhibit a high degree of consistency with the ground truth profiles, as evidenced by the majority of Pearson correlation coefficient values falling within the range of 0.8 to 1.0 across all samples (Fig. 2I–L). In this experiment, InferCNV demonstrated the second-best performance with single-cell Pearson correlation coefficient values ranging from 0.70 to 0.85. CopyKat, while exhibiting a slightly lower Pearson correlation, still achieved values above 0.7 for the majority of cells. However, SCEVAN’s estimation exhibited the least consistency with the ground truth copy profiles.

For a detailed robustness analysis of CopyVAE on synthetic data, see Supplementary Section S3. For the convergence results of the model, see Supplementary Section S4.

### 3.3 Performance evaluation on cancer dataset DCIS

**Figure 3.**
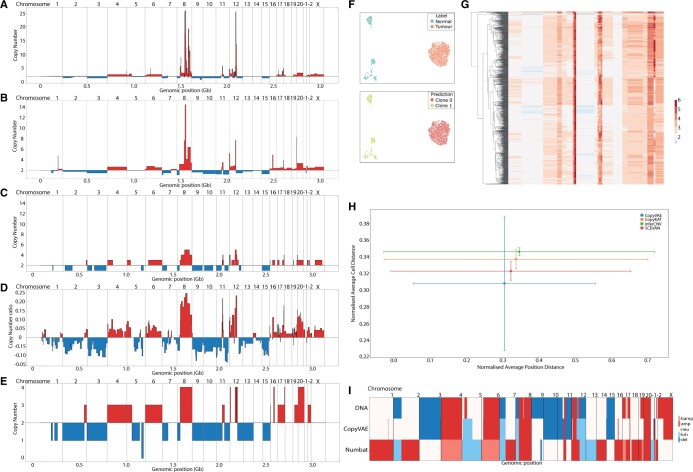
Experiment on Breast Cancer Dataset DCIS: (A) copy number profile called from bulk DNA-seq. (B) Copy number profile estimated by CopyVAE. (C) Copy number profile estimated by inferCNV. (D) Copy number profile estimated by CopyKAT. (E) Copy number profile estimated by SCEVAN. (F) UMAP of latent space representations: classification using cancer-specific marker genes (top) and CopyVAE clustering (bottom). (G) Heatmap of single-cell copy number profile estimated by CopyVAE. (H) Average cell distance versus average position distance, error bars represent the standard deviations. (I) LoH-aware copy number estimation of CopyVAE (middle) in comparison with Numbat (bottom).

We evaluated the accuracy of CopyVAE on a premalignant breast tumour sample sequenced using high-throughput 3ʹ scRNA-seq (10× Genomics). The dataset contains 1480 single cells, including both diploid and aneuploid ones. We used the copy number profile, obtained from a whole-genome bulk DNA sequencing analysis and provided by a previous study (Gao *et al.* 2021), as the ground truth reference in our benchmarking process. To assess CopyVAE’s performance, we compared it with four state-of-the-art methods: CopyKat, InferCNV, SCEVAN, and Numbat (Fig. 3A–E). While testing InferCNV, annotations were provided to indicate the cell ploidy, such as whether it is a tumour cell or a normal cell, which was derived from the clustering results of cancer-specific marker genes in the study by Gao *et al.* (2021). The same annotations were used while running Numbat. Comparing against this annotation, CopyVAE achieves a diploid cell detection accuracy of 99.18% (Fig. 3F).

CopyKat and InferCNV provide copy number ratios instead of integer copy numbers. In order to compare CopyKat, InferCNV, and CopyVAE on an equal basis, we converted CopyVAE integer copy number calls into log2 ratios. The tumour clone profile in CopyKat was estimated as the median copy number ratio of all identified tumour cells in each evaluation segment. For InferCNV, the median copy number ratios was calculated for genes, then mapped to genomic positions and aggregated for evaluated regions. The estimated copy numbers from the reference DNA copy number profile were compared using the evaluation metrics mentioned earlier for each method. Out of the four, CopyVAE showed the best performance with a Pearson correlation of 0.839, the lowest cosine distance (the complement of cosine similarity, i.e. 1 − cosine similarity), and the smallest positional average Manhattan and Euclidean distances as seen in Table 1. We evaluated single-cell copy number inferences (see the heatmaps in Fig. 3G and Supplementary Section S5) by comparing them to the ground truth profile. Figure 3H displays the normalized L1 distance between the ground truth profile and single-cell profiles estimated by CopyVAE, InferCNV, CopyKat, and SCEVAN, respectively. The vertical axis shows average cell distance, which is L1 distance averaged over cells and normalized by the number of positions. The horizontal axis shows average position distance, which is L1 distance averaged over positions normalized by the number of cells. The graph reveals that CopyVAE exhibits a low average deviation from reference DNA copy number while preserving aneuploid cell diversity.

**Table 1.** Performance on DCIS.

Method	Pearson correlation	Cosine distance	Avg Manhattan	Avg Euclidean
CopyKat	0.80341	0.19994	0.33445	0.00451
InferCNV	0.70101	0.30015	0.32399	0.00431
SCEVAN	0.81450	0.20491	0.30948	0.00420
CopyVAE	**0.83929**	**0.16702**	**0.18798**	**0.00292**

Bold values show the best results.

To compare with Numbat, the reference copy number profile of tumour cells was first converted to integer values and then a threshold of 2.0 was applied to identify amplifications and deletions. Copy numbers greater than the threshold were considered amplifications while those less than the threshold were considered deletions. The same threshold was also used to convert the CopyVAE results into the same measure as Numbat. We then utilized single-nucleotide polymorphism (SNP) variant allele frequency to detect loss of heterozygosity (LoH) regions. Genomic positions containing SNPs with variant allele frequency <0.1 or >0.9, and reported as neutral or deleted by CopyVAE, were considered LoH regions. Figure 3I presents the final comparison of the LoH-aware copy number profile generated by CopyVAE and Numbat. It is evident that CopyVAE concurs with Numbat’s inference in certain LoH regions in chr5, chr7, chr8, and chr12, while maintaining closer alignment to the reference DNA copy number profile overall.

### 3.4 Performance evaluation on cancer dataset CIIR

We performed Smart-Seq3 single-cell RNA sequencing (Hagemann-Jensen *et al.* 2020) and whole-exome sequencing on three samples referred to as BCSA1, BCSA2, and BCSA3, which are from three patients with breast cancer tumours. BCSA1 is a triple negative subtype and the other two are HER2+. Multiple regions were extracted from each of these tumours and both whole exome sequencing (WES) and Smart-seq3 single-cell RNA sequencing (scRNA-seq) was performed on each of them (except for BCSA1 where scRNA-seq was performed on a pool of cells from all tumour regions). Sample preparation and sequencing steps are already published in Jun *et al.* (2023) and the sequencing data of BCSA2 is uploaded to EGA with study ID EGAS00001006851. For scRNA processing, we used zUMIs (Parekh *et al.* 2018) version 2.9.7d using Ensembl version 105 annotation. After this procedure, a total of 1008 cells remained. In addition, as Supplementary Material to this article, we have made the scRNA-seq data of BCSA1 and BCSA3 publicly available for further analysis and validation.

**Figure 4.**
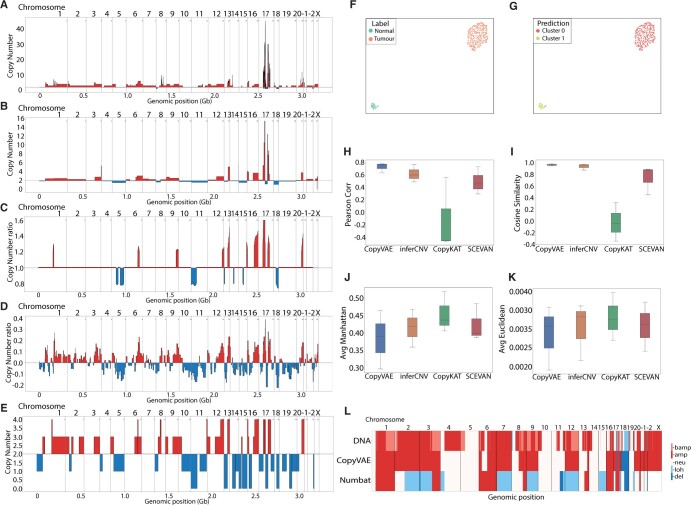
Experiment on Breast Cancer Dataset CIIR: (A) BCSA2 DNA copy number profile. (B) BCSA2 copy number profile estimated by CopyVAE. (C) BCSA2 copy number profile estimated by inferCNV. (D) BCSA2 copy number profile estimated by CopyKat. (E) BCSA2 copy number profile estimated by SCEVAN. (F) Cancer-specific marker genes based classification of BCSA2. (G) CopyVAE clustering of BCSA2. (H) Pearson correlation on all samples. (I) Cosine similarity on all samples. (J) Average Manhattan distance on all samples. (K) Average Euclidean distance on all samples. (L) Comparison of DNA copy number profile, CopyVAE estimation and Numbat estimation for BCSA2.

The experiments were conducted on three samples using CopyVAE, InferCNV, CopyKat, SCEVAN, and Numbat (Fig. 4A–G, Supplementary Section S6). During the preprocessing stage, cells were filtered based on the following criteria: total read counts <5000, expressed genes <2000, or mitochondrial gene percentage exceeding 20%. Mitochondrially encoded, HLA, and cell cycle genes were excluded from the input matrix. The resulting matrix underwent a normalization and binning procedure as described in the Section 2.1.

An additional step of cell type clustering is conducted specifically for running inferCNV. We used scVI for clustering the different cell types and identified tumour cell clusters using cancer-specific marker genes. The cells were annotated based on this classification. The resulting labels obtained from this procedure were used as input for the inferCNV analysis.

To obtain ground truth copy number profiles, the reads from whole exome sequencing were aligned to GRCh38 using BWA (Li and Durbin 2009) and then processed using GATK best practices for DNA (DePristo *et al.* 2011) for base score recalibration and duplicate removal. TitanCNA was run on this DNA data to call copy number profiles, hereafter this estimation of copy numbers is denoted as the DNA copy number profile. Aligning the copy number call outcomes of each method with the genomic positions of the copy number profile estimated by TitanCNA, we computed similarity metrics to quantify the disparity between the estimated copy number profile and the DNA copy number profile.

In the comparison with the DNA copy number profile, CopyVAE consistently demonstrated superior performance across all three samples (Fig. 4H–K). It exhibited the highest Pearson correlation coefficient: 0.769, 0.759, and 0.624 in samples BCSA1, BCSA2, and BCSA3 respectively; along with the lowest cosine distance and lowest average Euclidean distance (Table 2). Furthermore, CopyVAE achieved the lowest average Manhattan distance specifically in samples BCSA1 and BCSA3. Notably, CopyVAE exhibited consistent and reliable performance in terms of Pearson correlation and cosine similarity across all samples (Fig. 4H and I). In terms of overall estimation, InferCNV demonstrated the second-highest performance, particularly in samples BCSA1 and BCSA3. On the other hand, CopyKat encountered difficulties in accurately classifying diploid and aneuploid cells, leading to negative Pearson correlations in samples BCSA1 and BCSA3 (Table 2).

**Table 2.** Performance on CIIR.

Sample	Method	Pearson correlation	Cosine distance	Avg Manhattan	Avg Euclidean
BCSA1	CopyKat	−0.46475	1.04488	0.43689	0.00269
	InferCNV	0.75678	0.03094	0.35989	0.00215
	SCEVAN	0.28750	0.11611	0.39819	0.00240
	CopyVAE	**0.76902**	**0.02450**	**0.29771**	**0.00190**
BCSA2	CopyKat	0.55304	0.68872	0.40594	0.00325
	InferCNV	0.47993	0.13106	0.41855	0.00331
	SCEVAN	0.72336	0.13079	**0.38722**	0.00311
	CopyVAE	**0.75991**	**0.06190**	0.38925	**0.00306**
BCSA3	CopyKat	−0.45086	1.34038	0.51768	0.00395
	InferCNV	0.59044	0.04787	0.46716	0.00360
	SCEVAN	0.44829	0.55247	0.48306	0.00370
	CopyVAE	**0.62434**	**0.04100**	**0.46357**	**0.00357**

Bold values show the best results.

We conducted a comparative analysis between copyVAE and Numbat for sample BCSA2. In alignment with previous experimental settings, we transformed the integer DNA copy number profile of the tumour clone into distinct CNV categories, including balanced amplification, amplification, neutral, loss of heterozygosity (LoH), and deletions. We used the same conversion methodology to transfer the copy number estimated by CopyVAE into these categories. As illustrated in Fig. 4L, Numbat exhibited misclassification by identifying amplification as LoH in chromosomes 2, 3, 7, 9, and 12. In contrast, CopyVAE accurately predicted the presence of amplification. Since CopyVAE solely utilizes the UMI matrix for inference, it does not discern between balanced and unbalanced copy number amplification.

## 4 Discussion

In conclusion, this study presented a comprehensive analysis of copy number profiling using scRNA-seq data. With the proposed method CopyVAE, we successfully inferred copy number profiles at the single-cell level. We evaluated the performance of CopyVAE by comparing its results with ground truth copy number profiles obtained from DNA sequencing and whole-exome sequencing analyses. Our findings demonstrated that CopyVAE achieved accurate estimation of copy numbers, as evidenced by high Pearson correlation and cosine similarity values with the ground truth copy number profiles. We also quantified the estimation accuracy by calculating positional Manhattan and Euclidean distances, providing further insights into the fidelity of the inferred copy number profiles.

One notable limitation of CopyVAE is its heavy reliance on diploid cell identification for copy number inference. This dependence on the presence of normal cells in the sample can lead to decreased performance when there are no normal cells present. Note that the same limitation exists for the other methods as well. The resolution of our method may be constrained by the sequencing depth and the inherent noise in the data. It is worth noting that our method relies on specific assumptions and parameters that may not be universally applicable to all types of scRNA-seq data or experimental settings. These limitations should be considered when interpreting the results and drawing conclusions from our study.

The insights obtained from this study contribute to our understanding of somatic CNVs at the single-cell level, particularly in the context of cancer research. The CopyVAE approach, coupled with the outlined data processing pipeline, provides a valuable tool for researchers to explore copy number profiles with single-cell transcriptomic data. Future research could focus on further optimizing the algorithm’s performance and extending its application to other biological contexts.

Overall, this study demonstrates the potential of single-cell copy number profiling and provides a valuable framework for researchers to investigate genomic alterations and their implications in disease development and progression.

## Supplementary Material

btae284_Supplementary_Data

## Date availability

CopyVAE is implemented in Python and available at https://github.com/kurtsemih/copyVAE. DCIS data is retrieved from the Gene Expression Omnibus (GEO; GSE148673). CIIR data is uploaded to the European Genome-phenome Archive (EGA; Study ID EGAS00001006851).
