# Supplementary material for: CopyVAE: a variational autoencoder-based approach for copy number variation inference using single-cell transcriptomics
Source: Bioinformatics. 2024 Apr 27;40(5):btae284. doi: 10.1093/bioinformatics/btae284 (PMC11087824; doi:10.1093/bioinformatics/btae284)
Supplement: btae284_Supplementary_Data [file btae284_supplementary_data.pdf]

Supplementary for CopyVAE: a variational autoencoder-based approach for copy number variation inference using single-cell transcriptomics

1 Methods

1.1 Identification of diploid cells

The model is applied to count matrix X and latent representations for each cell are obtained. These representations are clustered via k-means clustering method. For each cluster, the auto-correlation score is computed as below:

$$\rho_m = \frac{E\big[(\boldsymbol{x_i} - \boldsymbol{\mu_m})(\boldsymbol{x_j} - \boldsymbol{\mu_m})^T\big]}{\sigma_m^2}$$

where ρ_m is the auto-correlation score, $\boldsymbol{\mu_m}$ is the mean expression profile, and σ_m^2 is the variance of cluster m. $\boldsymbol{x_i}$ and $\boldsymbol{x_j}$ denote the expression profiles of the cells i and j in cluster m.

Based on the assumption that normal cells exhibit lesser diversity in contrast to tumour cells (R Gao et al., 2021), the cluster with the greatest auto-correlation is identified as the diploid cells.

The baseline expression level of each bin is computed as the mean expression level of that bin among the identified diploid cells.

1.2 The CopyVAE model

The expression count x_{nb} of each bin b within each cell n undergoes a normalization process by referencing the bin b's specific baseline expression level. The normalized expression count then becomes the preliminary estimate of the copy number $\tilde{c}_{nb}$ of each bin b within each cell n. This preliminary estimate is referred to as the pseudo copy number and modelled as a sample drawn from a negative binomial (NB) distribution. Through a neural network, the unobserved latent random variable z_n associated with each cell n is mapped to the parameters characterizing this NB distribution, utilizing the actual copy number estimate c_{nb} for each bin b within each cell n.

The generative process for CopyVAE is described as follows: A standard multivariate normal prior is employed for the latent variable z_n, as in vanilla variational autoencoders (Kingma & Welling, 2013):

$$z_n \sim N(0, I)$$

The latent variable z_n is mapped to the unobserved copy number c_{nb} via a neural network f_c (NN2 in Figure 1 of the main document):

$$c_{nb} = f_c(z_n)$$

The latent variable z_n is also mapped to the dispersion parameter σ_{nb} of each bin b in each cell n, via another neural network f_d (NN3 in Figure 1 of the main document):

$$\sigma_{nb} = f_d(z_n)$$

Finally, the pseudo copy number $\tilde{c}_{nb}$ is modelled as a sample drawn from a NB distribution with the parameters c_{nb} and σ_{nb}:

$$\tilde{c}_{nb} \sim \mathrm{NB}\big(c_{nb}, \sigma_{nb}\big)$$

Both neural networks f_c and f_d consist of two fully connected layers and they share the weights for the first layer. A rectified linear unit (ReLU) activation function is utilized between the layers. All the networks employ dropout regularization and batch normalization. The output of the function f_c is passed through a sigmoid activation function and then scaled by a hyperparameter known as the maximum copy number. This scaling ensures that the copy number estimate remains within an upper limit. The output of f_d is subjected to the softplus activation function to assure the non-negativity of the inferred dispersion.

The posterior distribution of the latent variable $p(z_n|x_n)$ is approximated by training another neural network(NN1 in Figure 1 of the main document) using variational inference, similar to Kingma & Welling, (2013) and Lopez et al. (2018). The variational distribution $q(z_n|x_n)$ is chosen to be multivariate Gaussian with a diagonal covariance matrix. The mean and covariance of this distribution are obtained by applying a neural network to x_n. This network consists of one common fully connected layer and two separate additional layers: one for mean and one for covariance. A ReLU activation function is utilized between the layers. The common layer employs dropout regularization and batch normalization. The non-negativity of the inferred covariance is ensured by the exponentiation of the output of the covariance network.

The entire model is trained to optimize the evidence lower bound(ELBO):

$$log\, p(x|z) \geq E_{q(z|x)} log\, p(x|z) - D_{KL}\big(q(z|x)\big|\big|p(z)\big)$$

The first term on the right-hand side of the equation can be computed using the analytic expression for $p(x|z)$, the reparameterization trick, and low-variance Monte Carlo estimate of the expectation. The second term, on the

other hand, can be computed using the closed-form analytic expression for the Kullback–Leibler divergence, which is possible while both the variational and the prior distributions are Gaussian distributions.

The parameters of the entire model are updated using an Adam optimizer (Kingma & Ba, 2014) with the learning rate of 1e-3 and the epsilon of 1e-2. Supplementary Table 1 provides the complete list of the hyperparameters involved in the model. The selection of the number of layers and the number of hidden neurons were motivated by the other recent deep learning models on scRNA-seq data [4, 1, 3, 5]. The hyperparameters of the Adam optimizer, namely the learning rate and the epsilon were chosen by a grid search so that the held-out log-likelihood is maximized, which is a generalizable approach. Latent dimension was shown to not have a credible effect on the performance of CopyVAE (see Supplementary 3.1.2).

Supp. Table 1: Hyperparameter list of CopyVAE

**Data**	layers	hidden neurons	latent dimension	learning rate	ϵ
**Synthetic**	2	128	15	1e-3	1e-2
**DCIS**	2	128	20	1e-3	1e-2
**CIIR**	2	128	20	1e-3	1e-2

1.3 Segmentation and clone profile estimation

For the segmentation process, we used the PELT algorithm, an exact multiple changepoint detection method, as proposed by Killick et al. (2012). The goal of the segmentation is to identify a set of breakpoints $\tau_{1:m} = (\tau_1, \ldots, \tau_m)$ in a given sequence of data points $y_{1:n} = (y_1, \ldots, y_n)$ with an assumption $\tau_0 = 0$ and $\tau_{m+1} = n$, that minimises the total loss, as defined below:

$$\sum_{i=1}^{m+1} \left[\mathcal{L}\left(y_{(\tau_{i-1}+1):\tau_i}\right) + \beta\right]$$

where $\mathcal{L}$ is the loss function and β is a penalty constant to prevent overfitting. β is selected to be proportional to the product of the clone size and the logarithm of the number of positions. The loss function $\mathcal{L}$ for the segment between 2 breakpoints τ_a and τ_b is defined as the negative maximum log-likelihood function:

$$\mathcal{L}\left(y_{(\tau_a+1):\tau_b}\right) = -\max_{\theta} \sum_{i=\tau_a+1}^{\tau_b} \log P\left(y_i \mid \theta\right)$$

The observed data at a given location i, denoted by the variable y_i, is the set of copy numbers within a clone of size k at bin position i: $(c_{1i}, c_{2i}, c_{3i}, ...c_{ki})$,

where $c_{\upsilon i}$ is the copy number of bin i in cell υ. Assuming independence and identical distribution of cell copy numbers at a particular position, we can compute the likelihood of y_i, $P(y_i \mid \theta)$, as follows:

$$
\begin{aligned}
P(y_i \mid \theta) &= P(c_{1i}, c_{2i}, c_{3i} \ldots c_{ki} \mid \theta) \\
&= \prod_{\upsilon=1}^{k} P(c_{\upsilon i} \mid \theta)
\end{aligned}
$$

The PELT algorithm combines a recursive optimization method (Jackson et al., 2005) with a pruning technique, resulting in a computational cost for optimization that scales linearly with the number of cells. After determining the breakpoints for each tumour clone individually, single-cell copy numbers are obtained by computing the copy number of each segment between two breakpoints as the median value of the bin copy numbers within that segment. The single-cell copy number profiles are then aggregated by calculating the mean value of the segment copy numbers, resulting in the clone copy number profile.

1.4 Detection of loss of heterozygosity (LoH) regions

We utilise single-nucleotide polymorphism (SNP) variant allele frequency to detect loss of heterozygosity (LoH) regions. Because we assume that bulk DNA data is not necessarily available, we only use the scRNA-seq data. The process begins with selecting the SNPs with at least 5 percent population allele frequency in the 1000 Genomes project. Then for each of these SNPs the number of alternative and reference read counts in each cell is obtained using cell-snp-lite [2]. Then only those SNPs that have an allele frequency between 0.1 and 0.9 in the dataset are kept as heterozygous SNPs. After running CopyVAE, we exclude the normal cells and then if any bin has an average minor allele frequency less than 0.1 in the tumor cell population and is reported as neutral or deleted by CopyVAE, we call an LOH event for that bin.

2 Performance on synthetic dataset

Supp. Table 2: Performance on synthetic dataset

Sample	Method	Pearson correlation	Cosine distance	Avg Manhattan	Avg Euclidean
BM2-v0s0	CopyKat	0.71948	0.69694	-	-
	InferCNV	0.84477	0.03437	-	-
	SCEVAN	0.46507	0.05222	0.33869	0.00638
	CopyVAE	0.92559	0.00966	0.21739	0.00286
BM2-v0s1	CopyKat	0.74934	0.69894	-	-
	InferCNV	0.86158	0.06206	-	-
	SCEVAN	-0.88657	0.44769	1.35329	0.01888
	CopyVAE	0.92865	0.01401	0.28962	0.00380
BM2-v0s2	CopyKat	0.81992	0.55854	-	-
	InferCNV	0.89250	0.06645	-	-
	SCEVAN	-0.89464	0.37476	1.04461	0.01747
	CopyVAE	0.93031	0.01250	0.15788	0.00356
BM2-v0s3	CopyKat	0.76748	0.75606	-	-
	InferCNV	0.89002	0.06842	-	-
	SCEVAN	0.39569	0.09926	0.48369	0.00928
	CopyVAE	0.93436	0.01415	0.20481	0.00344
BM2-v0s4	CopyKat	0.77000	0.72131	-	-
	InferCNV	0.87388	0.05808	-	-
	SCEVAN	0.85280	0.02528	0.31399	0.00550
	CopyVAE	0.92116	0.01386	0.24549	0.00370
BM2-v0s5	CopyKat	0.73533	0.71542	-	-
	InferCNV	0.80520	0.06295	-	-
	SCEVAN	0.83669	0.02974	0.27085	0.00540
	CopyVAE	0.90806	0.01726	0.25003	0.00398
BM2-v0s6	CopyKat	0.78404	0.74580	-	-
	InferCNV	0.88606	0.04538	-	-
	SCEVAN	-0.92434	0.42019	1.22434	0.01774
	CopyVAE	0.94384	0.00866	0.24967	0.00311
BM2-v0s7	CopyKat	0.75970	0.72946	-	-
	InferCNV	0.82719	0.06360	-	-
	SCEVAN	0.38664	0.08663	0.86912	0.01200
	CopyVAE	0.92986	0.01304	0.27555	0.00394
BM2-v0s8	CopyKat	0.79272	0.58537	-	-
	InferCNV	0.87792	0.05788	-	-
	SCEVAN	-0.87259	0.41278	1.28058	0.01801
	CopyVAE	0.91028	0.01548	0.27555	0.00423
BM2-v0s9	CopyKat	0.75857	0.62024	-	-
	InferCNV	0.86286	0.06043	-	-
	SCEVAN	-0.89950	0.44709	1.32729	0.01832
	CopyVAE	0.90635	0.01814	0.32640	0.00434

3 Supplementary results on synthetic data

3.1 The effects of hyperparameters

3.1.1 Bin size

For the synthetic datasets BM2-v0s0 to BM2-v0s4, we ran CopyVAE with six different bin sizes ranging from 5 to 75. We quantitatively assessed the method's performance on clustering and copy number inference. To assess clustering, for each run we computed the silhouette score as the mean silhouette coefficient of all cells in the latent space. Supplementary Figure 1 shows that the silhouette score is maximum for the bin size of 25 genes. The explanation is that too small bin sizes do not strive enough to reduce the effects of noise and dropouts, whereas too large bin sizes end up averaging out some useful discriminative information in data.

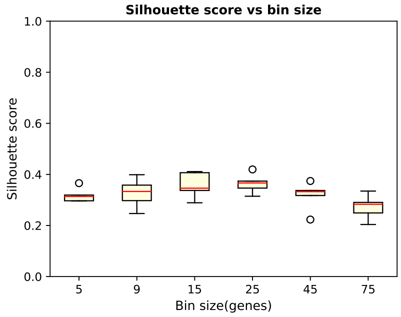

Supp. Fig. 1: Silhouette scores on the synthetic datasets BM2-v0s0 to BM2-v0s4, for bin sizes ranging from 5 to 75.

To assess copy number inference, we employed the evaluation metrics explained in section 3.1 of the main text. Supplementary Figure 2 shows that the performance of the method decreases slightly with the increased bin size. One should note that the choice of bin size does not depend on the performance, but on the robustness. Even though smaller bin sizes perform better, they may not be robust for the clustering process as evident from their relatively lower silhouette scores.

3.1.2 Latent dimension

For the synthetic datasets BM2-v0s0 to BM2-v0s4, we ran CopyVAE with six different latent dimension ranging from 5 to 50. We quantitatively assessed the method's performance using the evaluation metrics explained in the Section 3.1 of the main text. Supplementary Figure 3 shows that the latent dimension does not have an credible effect on the performance of CopyVAE.

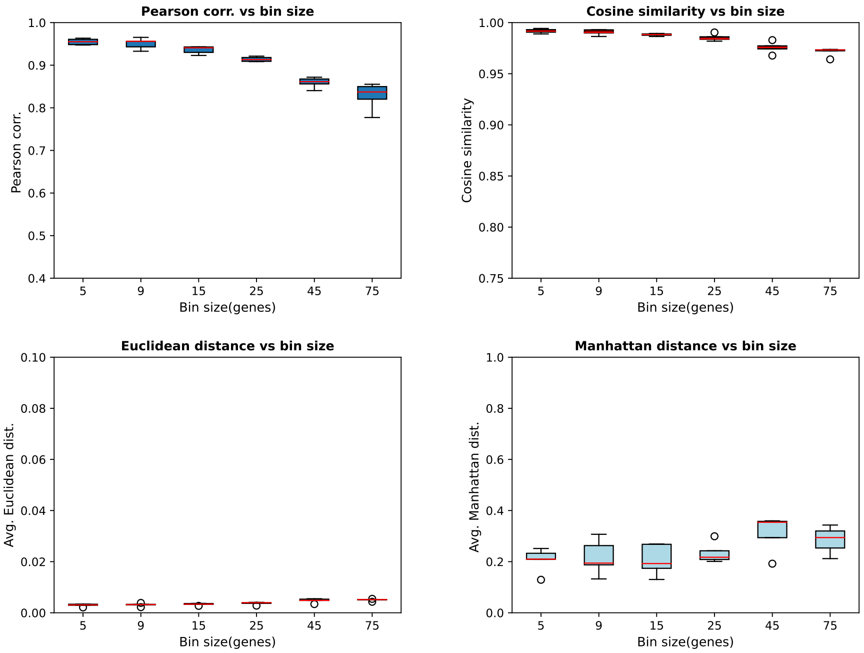

Supp. Fig. 2: The effect of bin size on CopyVAE's performance on the synthetic datasets BM2-v0s0 to BM2-v0s4, evaluated by the Pearson correlation (top-left), the cosine similarity (top-right), the average Euclidean distance (bottom-left), and the average Manhattan distance (bottom-right).

3.2 The effect of number of cells

Following the data generation pipeline explained in section 3.2.1 of the main text, we generated datasets consisting of different numbers of cells ranging from 200 to 1000. For each specific number of cells, we generated 5 different datasets (25 datasets in total). We evaluated the CopyVAE's performance on these datasets using the aforementioned evaluation metrics. Supplementary Figure 4 demonstrates that CopyVAE performs equally well for each number of cells.

3.3 The effect of tumor proportion

Following the data generation pipeline explained in section 3.2.1 of the main text, we generated datasets consisting of different tumour cell proportions: 0.6, 0.7, 0.8, 0.9. For each specific tumour proportion, we generated 5 different datasets (20 datasets in total). The CopyVAE's performance on these datasets was assessed using the previously mentioned evaluation metrics. Supplementary Figure 5 reveals that CopyVAE performs well for all the datasets, being independent of tumour proportion.

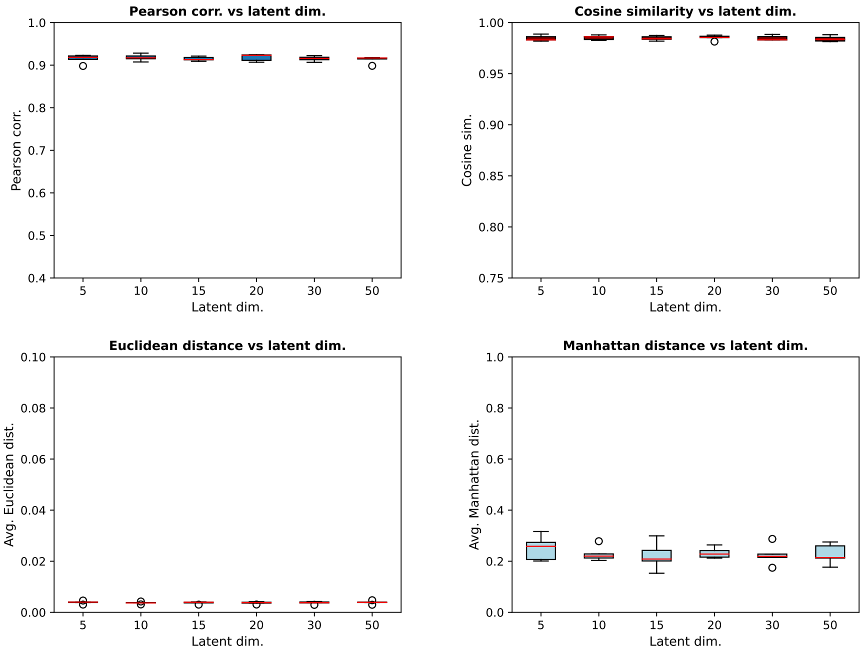

Supp. Fig. 3: The effect of latent dimension on CopyVAE's performance on the synthetic datasets BM2-v0s0 to BM2-v0s4, evaluated by the Pearson correlation (top-left), the cosine similarity (top-right), the average Euclidean distance (bottom-left), and the average Manhattan distance (bottom-right).

3.4 The effect of larger copy numbers

We introduced larger copy numbers up to 32 to the dataset BM2-v0s0, namely on a wide region in chromosome 9 and narrower regions in chromosome 15 (Supplementary Figure 6 - Top). We ran CopyVAE for this dataset keeping the maximum copy number hyperparameter as 6. The result showed that our method is quite robust for such a setting as well (Supplementary Figure 6 - Bottom). The amplified regions with copy numbers greater than 6 are estimated as having the maximum allowed copy number of 6. The other amplified and deleted regions are reconstructed correctly. A widespread low-level deletion artifact across the genome is observed. This artifact can be explained as caused by the extremely large copy numbers in the dataset affecting the baseline inference of CopyVAE. Due to those extremely amplified regions, normal cells' read counts are upscaled across the whole genome during normalization, giving a slightly overestimated baseline, which in turn leads to the aforementioned low-level deletion artifact. Besides these qualitative assessments, we also quantitatively evaluated the resulting profile comparing it to the ground truth. The comparison yielded in the Pearson correlation of 0.7372, the cosine similarity of 0.8964, the Euclidean distance of 0.0131, and the Manhattan distance of 0.6502, producing evidence

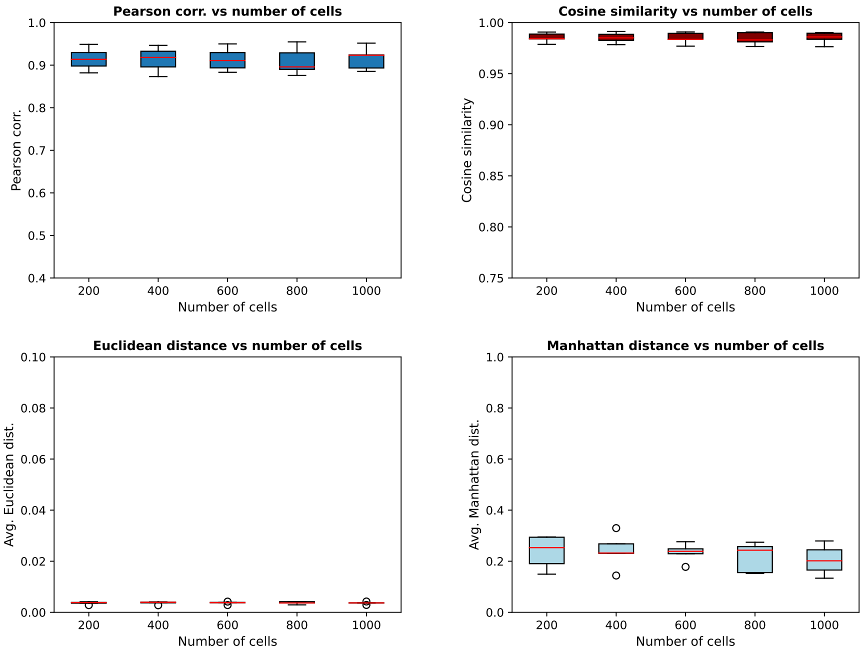

Supp. Fig. 4: The effect of number of cells on CopyVAE's performance, evaluated by the Pearson correlation (top-left), the cosine similarity (top-right), the average Euclidean distance (bottom-left), and the average Manhattan distance (bottom-right).

of the robustness of CopyVAE against extremely large copy numbers.

3.5 The effect of correlation among CNVs

We simulated correlated CNVs using CNAsim [6], which is a recent method for an improved generation of single-cell CNVs via simulating an exponentially growing tumor population under neutral coalescence. CNVs generated by CNAsim possess complex clonal correlations. We tested CopyVAE on a synthetic dataset having such correlated CNVs (Supplementary Figure 7 - Top) and the results showed that those correlations do not appreciably affect the performance of CopyVAE (Supplementary Figure 7 - Bottom). We further evaluated the performance of CopyVAE by comparing it against the ground truth profile using the evaluation metrics. The comparison yielded in the Pearson correlation of 0.9079, the cosine similarity of 0.9799, the Euclidean distance of 0.0047, and the Manhattan distance of 0.4079, submitting evidence of the robustness of CopyVAE for correlated CNVs.

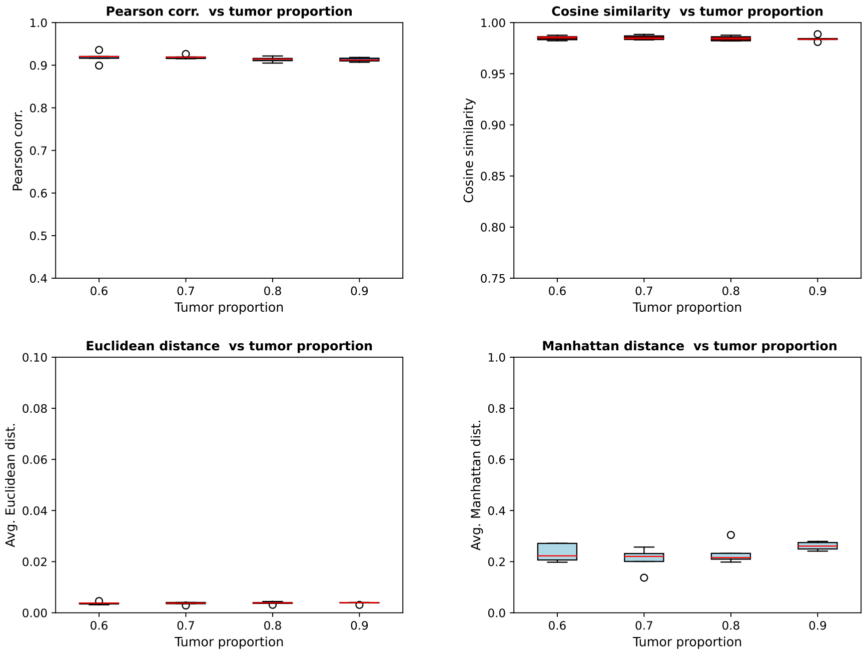

Supp. Fig. 5: The effect of tumour proportion on CopyVAE's performance, evaluated by the Pearson correlation (top-left), the cosine similarity (top-right), the average Euclidean distance (bottom-left), and the average Manhattan distance (bottom-right).

3.6 The effect of number of clones

We simulated a dataset consisting of 1000 cells and four clones, using CNAsim [6] to generate CNVs. We applied CopyVAE to this dataset. Supplementary Figure 8 shows that CopyVAE successfully detected the normal cells and the different clones. Compared against the ground truth labels, CopyVAE achieved a clustering accuracy of 0.947. CopyVAE performed well for the copy number profile estimation of each clone as well. As seen from Supplementary Figure 9, the profiles estimated by CopyVAE are in line with the ground truth profiles. Supplementary Table 3 shows also quantitatively that CopyVAE exhibits a stable performance among the different clones.

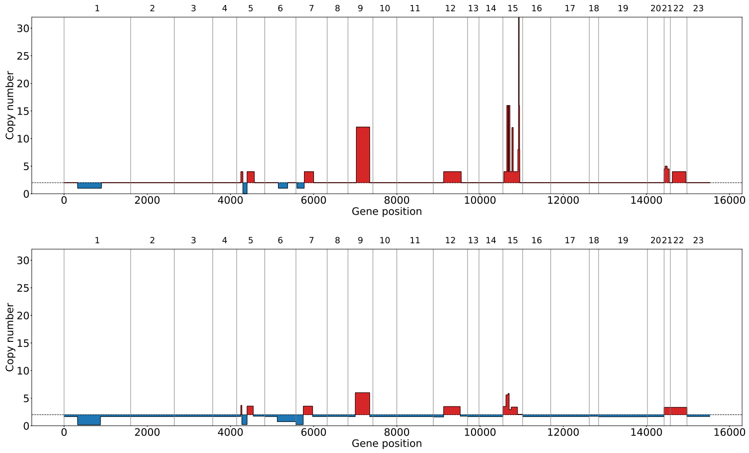

Supp. Fig. 6: The performance of CopyVAE for data having extremely large copy numbers: Top: Ground truth copy number profile. Bottom: Copy number profile estimated by CopyVAE.

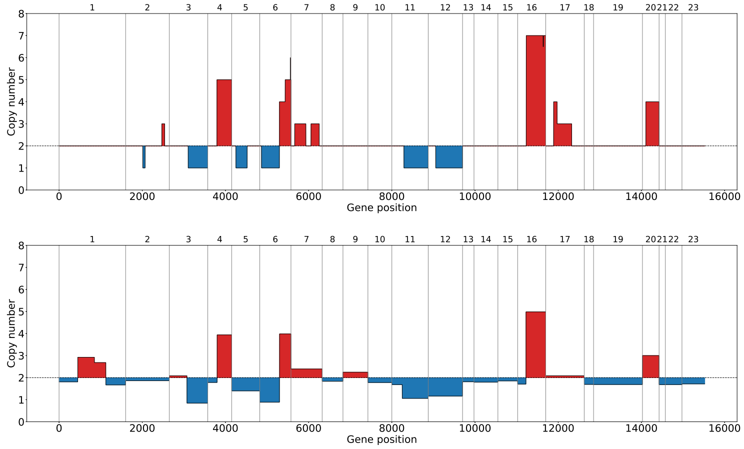

Supp. Fig. 7: The performance of CopyVAE for data having correlated CNVs: Top: Ground truth copy number profile. Bottom: Copy number profile estimated by CopyVAE.

Supp. Table 3: CopyVAE's performance on mutliple clones.

**CopyVAE**	Pearson correlation	Cosine distance	Avg Manhattan	Avg Euclidean
Clone1	0.90537	0.01954	0.32569	0.00328
Clone2	0.94376	0.02177	0.34923	0.00358
Clone3	0.92575	0.01698	0.25957	0.00323
Clone4	0.85277	0.02746	0.29377	0.00400

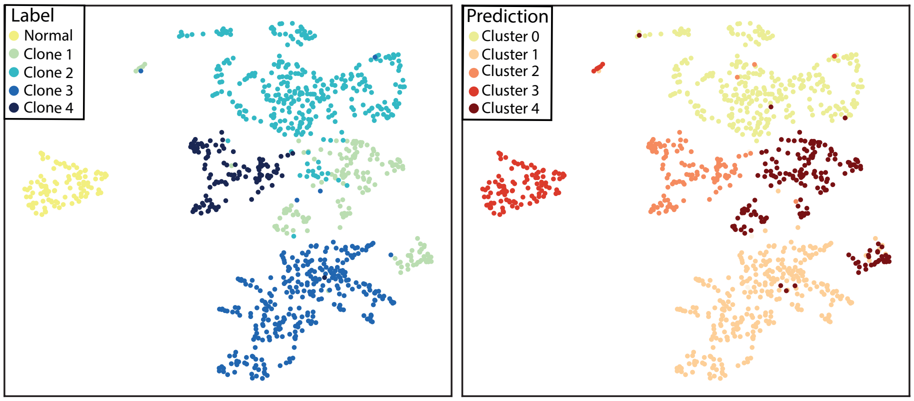

Supp. Fig. 8: UMAP of latent space representations for data having four clones: ground truth clusters (left) and CopyVAE clustering (right).

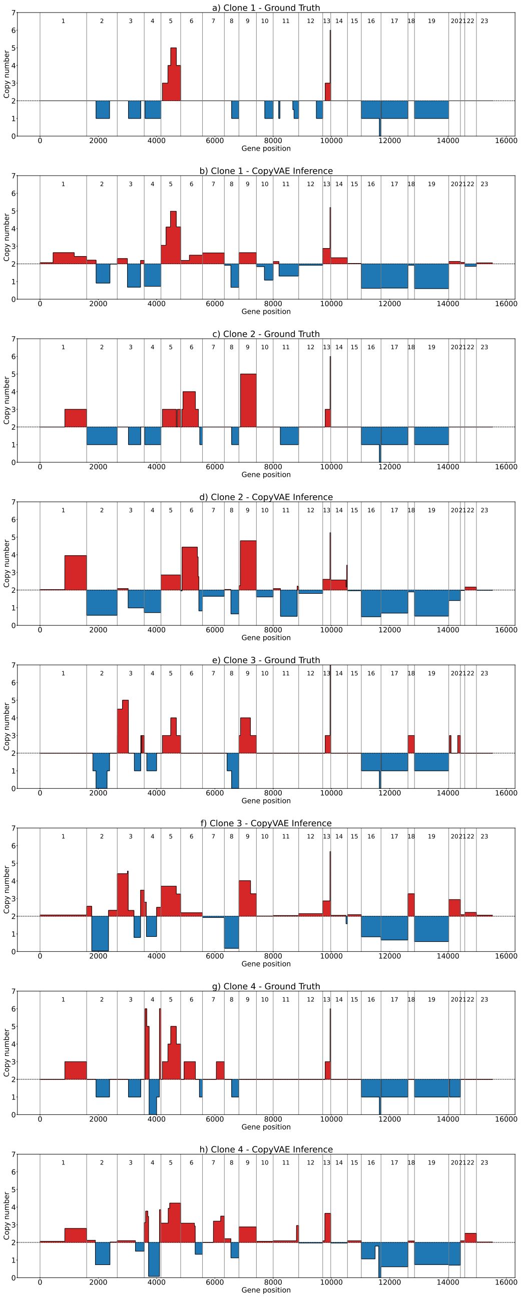

Supp. Fig. 9: The performance of CopyVAE for data having four clones: a) Ground truth copy number profile of clone 1. b) Clone 1's copy number profile estimated by CopyVAE. c) Ground truth copy number profile of clone 2. d) Clone 2's copy number profile estimated by CopyVAE. e) Ground truth copy number profile of clone 3. f) Clone 3's copy number profile estimated by CopyVAE. g) Ground truth copy number profile of clone 4. h) Clone 4's copy number profile estimated by CopyVAE.

4 Convergence of the model

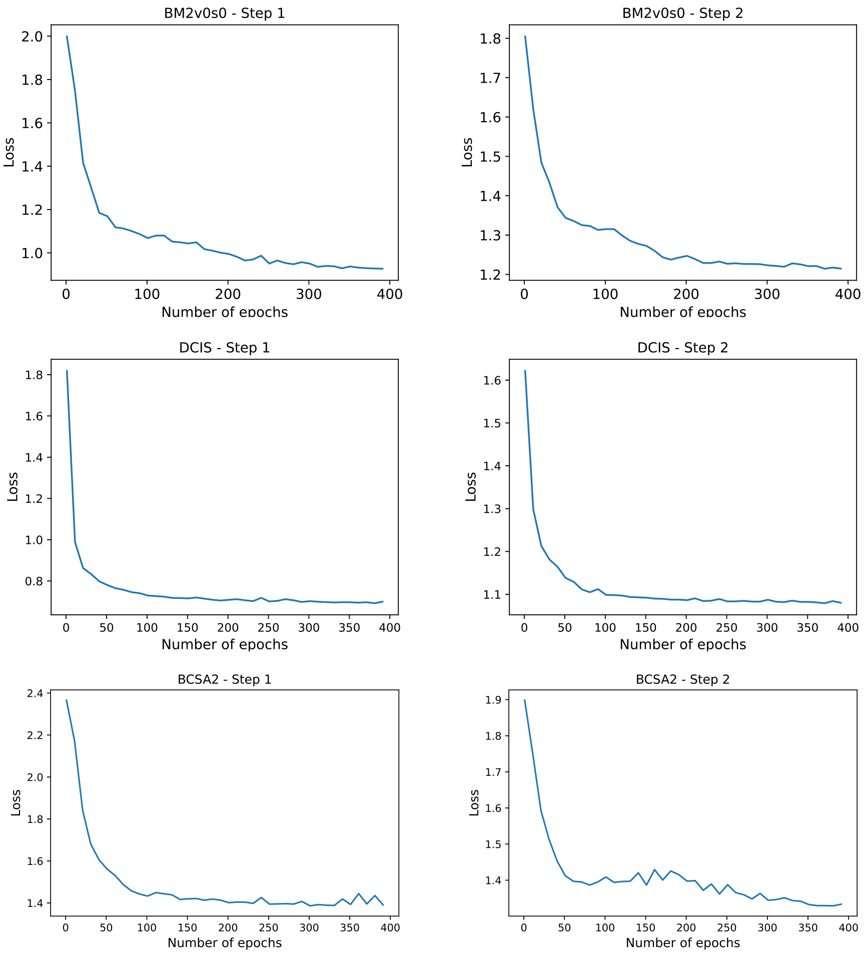

Supp. Fig. 10: Loss curves of CopyVAE for the datasets BM2-v0s0, DCIS, and BCSA2.

5 Supplementary figure for DCIS

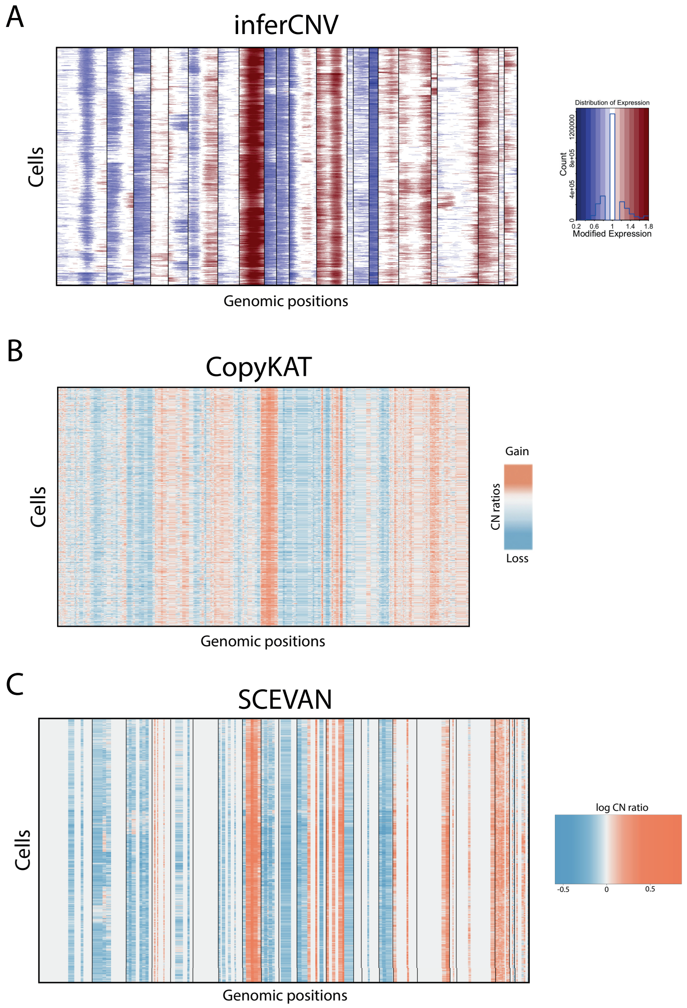

Supp. Fig. 11: Heatmaps of DCIS's single-cell copy number profile estimated by a) inferCNV, b) CopyKAT, c) SCEVAN.

6 Supplementary figures for BCSA1 and BCSA3

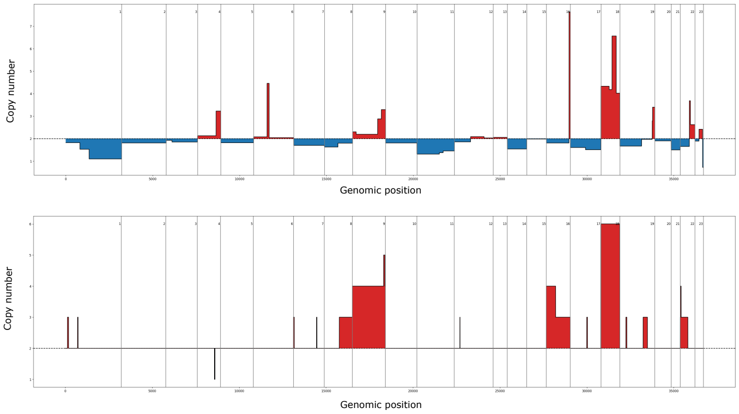

Supp. Fig. 12: Copy number profile of sample BCSA1: Top: Copy number profile estimated by CopyVAE. Bottom: Copy number profile called from bulk DNA-seq.

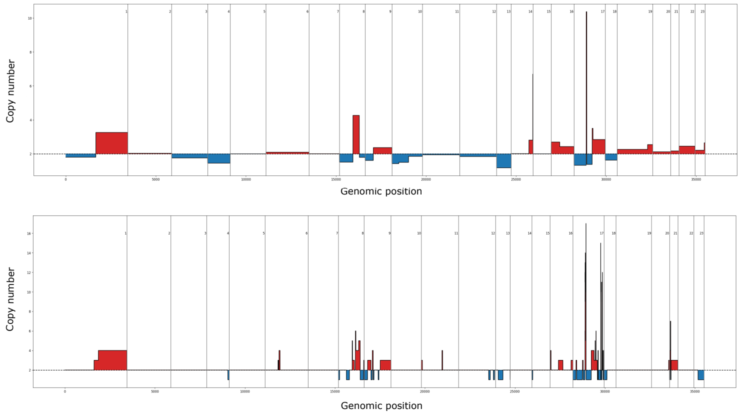

Supp. Fig. 13: Copy number profile of sample BCSA3: Top: Copy number profile estimated by CopyVAE. Bottom: Copy number profile called from bulk DNA-seq.

References

[1] Christopher Heje Grønbech, Maximillian Fornitz Vording, Pascal Timshel, Casper Kaae Sønderby, Tune Hannes Pers, and Ole Winther. scvae: Variational auto-encoders for single-cell gene expression data. *bioRxiv*, 2019.

[2] Xianjie Huang and Yuanhua Huang. Cellsnp-lite: an efficient tool for genotyping single cells. *Bioinformatics*, 37(23):4569–4571, 2021.

[3] Romain Lopez, Baoguo Li, Hadas Keren-Shaul, Pierre Boyeau, Merav Kedmi, David Pilzer, Adam Jelinski, Ido Yofe, Eyal David, Allon Wagner, Can Ergen, Yoseph Addadi, Ofra Golani, Franca Ronchese, Michael I Jordan, Ido Amit, and Nir Yosef. DestVI identifies continuums of cell types in spatial transcriptomics data. *Nature Biotechnology*, 40(9):1360–1369, September 2022.

[4] Romain Lopez, Jeffrey Regier, Michael B Cole, Michael I Jordan, and Nir Yosef. Deep generative modeling for single-cell transcriptomics. *Nature methods*, 15(12):1053–1058, 2018.

[5] Dongfang Wang and Jin Gu. Vasc: dimension reduction and visualization of single cell rna sequencing data by deep variational autoencoder. *bioRxiv*, 2017.

[6] Samson Weiner and Mukul S Bansal. CNAsim: improved simulation of single-cell copy number profiles and DNA-seq data from tumors. *Bioinformatics*, 39(7), July 2023.
